# Sumoylation in Synaptic Function and Dysfunction

**DOI:** 10.3389/fnsyn.2016.00009

**Published:** 2016-04-28

**Authors:** Lenka Schorova, Stéphane Martin

**Affiliations:** Institut de Pharmacologie Moléculaire et Cellulaire, Centre National de la Recherche Scientifique (UMR7275), University of Nice—Sophia-Antipolis, Laboratory of Excellence “Network for Innovation on Signal Transduction, Pathways in Life Sciences”Valbonne, France

**Keywords:** synapse, post-translational modification, sumoylation, desumoylation, SUMO

## Abstract

Sumoylation has recently emerged as a key post-translational modification involved in many, if not all, biological processes. Small Ubiquitin-like Modifier (SUMO) polypeptides are covalently attached to specific lysine residues of target proteins through a dedicated enzymatic pathway. Disruption of the SUMO enzymatic pathway in the developing brain leads to lethality indicating that this process exerts a central role during embryonic and post-natal development. However, little is still known regarding how this highly dynamic protein modification is regulated in the mammalian brain despite an increasing number of data implicating sumoylated substrates in synapse formation, synaptic communication and plasticity. The aim of this review is therefore to briefly describe the enzymatic SUMO pathway and to give an overview of our current knowledge on the function and dysfunction of protein sumoylation at the mammalian synapse.

## Introduction

As the mammalian brain develops, crucial sequential processes take place for a functional neuronal circuitry to be established. These processes are as follows: embryonic neurogenesis that gives rise to neuronal cells from the progenitors within the neural tube; migration of these newly born neurons to their destination area that is followed by maturation and formation of interneuronal connections. The spatiotemporal regulation of these processes, which results in the formation and the stabilization of synaptic connections, participates in the shaping of a physiologically active and functional brain circuitry. The formation of a mature functional synaptic contact starts with an axonal outgrowth until the growth cone reaches a target neuron. The axonal growth cone transforms into a presynaptic terminal that is characterized by the presence of neurotransmitter-filled synaptic vesicles and faces the postsynaptic area i.e., the dendritic spine, which is enriched in neurotransmitter receptors. The functional synapse is capable of integrating an electrical signal into biochemical changes, a process referred to as synaptic transmission. Importantly, the term “synaptic plasticity” regroups a wide range of molecular mechanisms that allow the modification of the strength and efficacy of synaptic transmission, and thereby underpin the ability of the brain to respond to environmental changes and/or experiences, and consequently underlie cognitive functions.

The molecular composition and organization of a mature synapse is incredibly complex. It has been estimated that “an average” synapse contains 300,000 proteins (Wilhelm et al., 2014). As these proteins mediate synaptic transmission and plasticity their interactions must be regulated both in time and space and this is mostly achieved by posttranslational modifications (PTMs). Accordingly, it is widely accepted that most types of plasticity are expressed through changes in the number of postsynaptic glutamate receptors and these changes are regulated by PTMs (for a comprehensive review, see Yokoi et al., 2012). For instance, both CaMKII and PKC phosphorylation of the GluA1 subunit of AMPA receptors (AMPARs) increase single-channel conductance of AMPARs leading to expression of long-term potentiation (LTP) in the hippocampus. In addition, previous studies have reported that PKC-phosphorylation of the AMPAR subunit GluA2 regulates its protein-protein interactions in the cerebellum leading to the expression of an activity-dependent long-term decrease in synaptic strength known as long-term depression (LTD; Matsuda et al., 2000). It has also been shown that the synaptic function can be regulated via other PTMs. Ubiquitination is a reversible PTM that can direct target proteins for degradation through the ubiquitin proteasome system (UPS). Bingol and Schuman (2006) reported that proteasome constituents and ubiquitin moiety are present in dendrites and upon neuronal activation the dendritic UPS moves into spines to shape the synaptic protein composition and subsequently the synaptic function. Interestingly, sumoylation has recently emerged as an essential PTM in the central nervous system (CNS) that profoundly alters protein activity, stability and subcellular localization, controls protein-protein interactions, and is important for the brain development and the regulation of synaptic communication (for recent reviews see Gwizdek et al., 2013; Henley et al., 2014). Moreover, perturbations in neuronal sumoylation have been implicated in numerous pathological conditions (reviewed in Dorval and Fraser, 2007; Martin et al., 2007b; Martin, 2009; Krumova and Weishaupt, 2012; Lee et al., 2013; Henley et al., 2014).

## The Enzymatic Machinery of Small Ubiquitin like Modifiers

Sumoylation is an evolutionarily conserved enzymatic pathway, analogous to the ubiquitination process, which covalently and reversibly conjugates a small protein of ~100 amino acids, called Small Ubiquitin-like Modifier(SUMO, ~11 kDa), to lysine residues of target proteins (Matunis et al., 1996; Mahajan et al., 1997).

Four SUMO paralogs have been identified in humans until now. SUMO1–3 are ubiquitously expressed (Hay, 2005; Geiss-Friedlander and Melchior, 2007) whereas SUMO4 expression seems restricted to the spleen, the kidney and the lymphatic nodes (Bohren et al., 2004; Guo et al., 2004). SUMO2 and SUMO3 are nearly identical except three additional N-terminal residues within the SUMO3 sequence; therefore they are generally referred to as SUMO2/3. On the contrary, SUMO1 shares only ~50% sequence identity with SUMO2/3. SUMO1 and SUMO2/3 modify an overlapping set of target proteins; but they differ in their properties and subcellular abundance with the amount of free available SUMO2/3 being much larger that of SUMO1.

In most cases, the SUMO-targeted lysine resides within a specific consensus site defined as ψ-K-x-D/E, where ψ corresponds to a large hydrophobic residue, K stands for lysine, x is any amino acid, and D/E are glutamate and aspartate acidic residues respectively (Rodriguez et al., 2001; Sampson et al., 2001). Importantly, not all consensus sequences are sumoylated and not all SUMO-target proteins are modified within this particular motif. Several additional sumoylation sites were identified, which revealed that the sequences flanking the target lysine residue are critical to determine whether a site can be SUMO-modified or not (reviewed in Flotho and Melchior, 2013; Henley et al., 2014). It is also important to note that many of the lysine residues contained within SUMO consensus sites are reported as not sumoylated. However, in most cases the determination of such sumoylation status was achieved in basal unstimulated conditions. Therefore, caution should be taken to definitively state that a given protein is not a SUMO substrate since only a small proportion of a specific protein is sumoylated at steady state (Hay, 2005).

**Figure 1:**
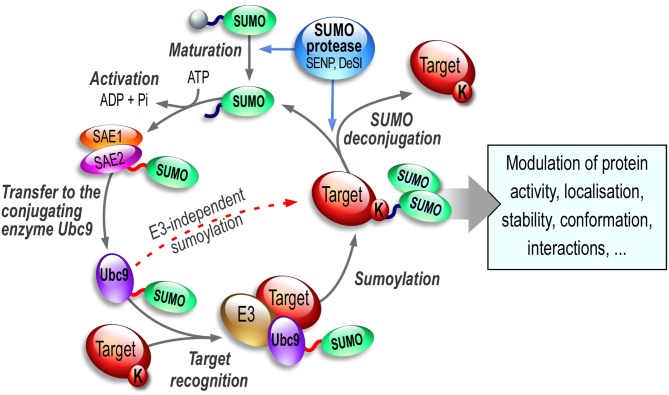
**The SUMO enzymatic pathway.** SUMO paralogs are synthesized as inactive precursors that are first matured by the hydrolase activity of specific desumoylases called SENPs. SUMO activation is an ATP-dependent step leading to formation of a thioester bond between the SUMO-activating subunit SAE2 of the E1 enzymatic heterodimer SAE1/SAE2 and the activated SUMO. SUMO is then transferred onto the active (C93) cysteine residue of Ubc9, the sole E2-conjugating enzyme of the SUMO system. Ubc9 is able to catalyze the sumoylation reaction of the target lysine residue on the substrate either directly or in combination with one of the existing SUMO E3 ligases. Importantly, sumoylation is readily reversible and sumoylated proteins can be efficiently desumoylated via the isopeptidase activity of a variety of SUMO proteases including SENPs, DeSI1/2 and/or USPL1.

The sumoylation/desumoylation cycle (Figure 1) starts with the cleavage of inactive SUMO precursor proteins by the hydrolase activity of the SENtrin-specific Protease (SENP, Mukhopadhyay and Dasso, 2007; Hickey et al., 2012) enzymes so the C-terminal di-glycine motif on SUMO is uncovered for conjugation. Mature SUMOs are then activated by the SAE1 and SAE2 (SUMO-activating enzyme subunit 1 and 2; also named AoS1/Uba2 in rodents) heterodimer complex in an ATP-dependent manner. Afterwards, SUMO conjugation to target substrate proteins is carried out by the sole conjugating enzyme of the SUMO system, Ubc9 (Figure 1). This conjugation step can be achieved either directly or in combination with an E3 SUMO ligase (Bernier-Villamor et al., 2002). These E3 proteins assist the sumoylation step either by bringing the substrate and the SUMO-Ubc9 in close proximity or by enhancing the transfer rate of SUMO onto the substrate (reviewed in Gareau and Lima, 2010; Flotho and Melchior, 2013). In the brain, the mechanisms, by which these E3 SUMO ligases operate and how they participate in the SUMO process to enhance sumoylation in neurons, are still largely unknown. However recent evidence suggests that these E3 ligases may be extremely important to tightly regulate the synaptic function.

The sumoylation/desumylation cycle (Figure 1) is highly dynamic and must be tightly controlled as it drastically influences the function of many proteins targeted by this PTM. Despite a covalent SUMO binding, sumoylation is a reversible process due to the isopeptidase activity of specific enzymes (see Hickey et al., 2012, for a comprehensive review on SUMO proteases). The desumoylation enzymes allow the removal of the SUMO moieties from modified substrates leaving non-sumoylated proteins and matured SUMOs available to re-enter the SUMO pathway. Several SUMO proteases effectively mediate this desumoylation process. In humans, six SENP proteases have been described (SENP1, 2, 3, 5, 6 and 7). These desumoylation enzymes differ in their subcellular localization and SUMO selectivity (Hickey et al., 2012). Recently, several additional SUMO proteases have been identified, DeSumoylating Isopeptidase 1 and 2 (DeSI1 and DeSI2; Shin et al., 2012) and USPL1 (Ubiquitin-Specific Protease-Like 1; Schulz et al., 2012). These SUMO proteases together with the SUMO-conjugating pathway convey an essential role to allow the dynamic equilibrium between the sumoylated and desumoylated state of many proteins. Since sumoylation participates in the regulation of many proteins involved in essential developmental processes and synaptic functions, dysregulation of the sumoylation/desumoylation balance may directly link the SUMO process to a number of pathophysiological conditions (see thereafter, “Sumoylation in Synaptopathies” Section).

## Sumoylation in Brain Development, Neuronal Maturation and Synapse Formation

### Sumoylation in Brain Development

Sumoylation acts throughout the neuronal cell to dynamically modulate protein function and consequently SUMO enzymatic machinery members present a widespread subcellular distribution including the nucleus (Martin et al., 2007a; Loriol et al., 2012; Wang et al., 2012; Hasegawa et al., 2014), the mitochondrial surface (Guo et al., 2013), the dendritic shaft and both pre- and postsynaptic elements (Martin et al., 2007a; Feligioni et al., 2009; Loriol et al., 2012, 2013, 2014; Gwizdek et al., 2013; Luo et al., 2013; Hasegawa et al., 2014).

To date, three separate studies have examined the spatiotemporal distribution of the sumoylation machinery members in the developing rodent brain with consistent results (Watanabe et al., 2008; Loriol et al., 2012; Hasegawa et al., 2014). The expression levels of both Ubc9 and SUMO1 mRNA are developmentally regulated in the rat brain, with higher expression levels before birth (Watanabe et al., 2008). Only recently, we reported that the protein expression levels of sumoylated proteins, the SUMO-activation enzyme SAE1, the SUMO-conjugating Ubc9 and the two desumoylation enzymes SENP1 and SENP6 are developmentally regulated in the rat brain (Loriol et al., 2012). SUMO1/2/3-conjugated protein levels are at their maximum at embryonic day 12, followed by a slow decrease until birth. SUMO1-modified protein levels progressively decrease until the adult stage. However, a second increasing phase occurs for SUMO2/3-ylated substrates just after birth. Interestingly, while the overall sumoylation slowly decreases after birth reaching relatively low levels in the adult brain, there are progressively more SUMO substrates within synaptic compartments (Loriol et al., 2012). The relative accumulation in synaptic SUMO substrates in aged rat brain is also consistent with an enrichment of the sumoylation enzymes AoS1 and Ubc9 in dendritic spines of fully mature rat neurons.

More recently, Hasegawa et al. (2014) combined immunohistochemical and immunoblot analyses on mouse brain at various developmental stages and also showed a developmental distribution of all SUMO moieties and the SUMO-conjugating enzyme Ubc9. During embryonic development, sumoylation occurred in the nucleoplasm of nestin-positive neural stem cells. Although the total amount of SUMO-modified proteins decreased during postnatal mouse brain development similar to the developing rat brain (Loriol et al., 2012), a persistent accumulation of SUMO2/3 was detected in neural progenitor populations in neurogenic regions throughout life (Hasegawa et al., 2014). In addition, a strong SUMO1-immunoreactivity was observed in large projection neurons in the brainstem suggesting that SUMO1- and SUMO2/3-modified proteins exert specific functions in the mouse brain.

The abundance and distribution of the sumoylation machinery play a critical role during embryonic and postnatal development. For instance, it was previously thought that SUMO2 and SUMO3 paralogs act in a totally redundant manner. Wang et al. (2014) recently revealed that SUMO2 is the predominantly expressed isoform in early embryonic stages of mouse development. Indeed, SUMO3-KO mice are viable while SUMO2 deficiency in mice leads to severe developmental delay and embryonic lethality, which strongly suggests that the spatiotemporal expression of these SUMO moieties, and not their functional differences (the two paralogs being almost identical), is a critical factor during the brain development.

Fu et al. (2014) investigated the role of SENP2 in the brain development by engineering a mouse model that expressed SUMO-protease activity-deficient SENP2 in neural progenitors. The authors showed that SENP2 is indispensable for the brain development. Indeed, SENP2 loss of function evoked an increase in neuronal sumoylation levels eventually leading to a robust post-natal neurodegeneration resulting in paralysis and death of the mice by three weeks of age (Fu et al., 2014). They also demonstrated that this neurodegeneration is the consequence of the hyper-sumoylation of Drp1 (Dynamin-related protein 1), which promoted its enhanced association with mitochondria and their subsequent fragmentation leading to neuronal apoptosis. Altogether, these data confirmed the importance of a controlled balance between the sumoylation and desumoylation state of a protein in the developing brain.

### Sumoylation in Neuronal Maturation and Synapse Formation

We have reported, that the sumoylation enzymes AoS1, Ubc9 as well as the SUMO proteases SENP1 and SENP6 were differentially redistributed in pre- and postsynaptic areas during neuronal maturation (Loriol et al., 2012). We further showed that the redistribution of the sumoylation machinery in and out of synapses is also observed upon neuronal depolarization and that this enzymatic redistribution impacts the synaptic levels of sumoylation (Loriol et al., 2013). Altogether, these data suggested that the SUMO process and the involved SUMO targets are not only important for the brain development but also for the maturation of neuronal cells and consequently for synaptic function.

#### MeCP2 (Methyl CpG Binding Protein 2)

Hundreds of mutations within the MeCP2 gene, which is located on the X-chromosome, have been linked to neurodevelopmental disorders, most frequently to Rett syndrome in females but also to some forms of autism, and schizophrenia. The Rett syndrome is behaviorally characterized by a developmental stagnation in early childhood associated with severe cognitive impairment and autistic features, the loss of spoken language and hand use. The encoded MeCP2 protein is a DNA-binding protein expressed ubiquitously and acts as a transcriptional repressor that fulfils key roles during the synaptic development (Guerrini and Parrini, 2012). Sumoylation of MeCP2 regulates its interaction with the transcriptional repression complex HDAC1/2 and preventing sumoylation in MeCP2 at the K223 residue leads to abnormal gene expression and impaired synaptic density (Cheng et al., 2014).

More recently, Tai et al. (2016) reported that MeCP2 is modified on different lysine residues e.g., K353 and K412, but failed to detect the K223 sumoylation. They showed that phosphorylation is required for MeCP2 sumoylation and that the SUMO E3 ligase PIAS actively participates in MeCP2 modification. They also elegantly demonstrated that MeCP2 sumoylation in the hippocampus is induced by several factors including the activation of NMDA receptors (NMDARs), the Insulin-like growth factor (IGF-1) and the Corticotropin-Releasing Factor (CRF) revealing a previously unsuspected activity-dependent regulation of MeCP2 sumoylation. Importantly, preventing MeCP2 sumoylation using the non-sumoylatable K412R MeCP2 mutant leads to a decrease in its DNA binding ability whereas a MeCP2-SUMO1 fusion significantly increases its DNA binding capabilities (Tai et al., 2016). Altogether, these data reinforce the idea that MeCP2 sumoylation is essential to its function and acts as a central regulator of MeCP2 function in the brain.

#### MEF2 Proteins (Myocyte-Enhancer Factor 2)

The establishment of functional synaptic circuits relies on the concomitant activity-dependent formation and elimination of synapses. MEF2 members form a family of four evolutionarily conserved transcriptional factors (MEF2A, B, C and D) that were first identified in muscle differentiation. They are also expressed throughout the brain including areas involved in cognitive functions (cortex, hippocampus, amygdala, striatum; reviewed in Rashid et al., 2014). Mutations within MEF2 genes have been directly linked to various pathological conditions including epilepsies, autism, and some neurodegenerative disorders (Flavell and Greenberg, 2008; Li et al., 2008; Yin et al., 2012) suggesting that these brain diseases could be triggered by abnormal MEF2-dependent gene transcription programs. Notably, MEF2 s are involved in several important neurodevelopmental processes including cell differentiation, dendritic morphogenesis, synapse formation, pruning and synaptic plasticity.

MEF2 activities are regulated through several PTMs including acetylation (Grégoire and Yang, 2005; Shalizi et al., 2007), phosphorylation (Flavell et al., 2006; Kang et al., 2006) and sumoylation (Grégoire and Yang, 2005; Zhao et al., 2005; Shalizi et al., 2006, 2007; Lu et al., 2013). A decade ago, Shalizi et al. (2006) investigated the SUMO-dependent repression of MEF2A in the developing cerebellar cortex. They demonstrated that there is an activity-dependent switch from a sumoylated MEF2A at the lysine 403 to its acetylated state leading to MEF2A activation and inhibition of dendritic claw differentiation and consequently to synapse disassembly (Shalizi et al., 2006). They used overexpression and knockdown strategies to show that PIASx is a MEF2 SUMO-E3 ligase linking this E3 protein to postsynaptic dendritic claw morphogenesis in the cerebellar cortex and confirming the essential role of protein sumoylation in the developing brain (Shalizi et al., 2007). MEF2A was also reported to be sumoylated both *in vitro* and *in vivo* at the lysine 395 residue and the E3 SUMO ligase PIAS1 enhances its sumoylation and subsequently decreases its transcriptional activity (Riquelme et al., 2006).

More recently, the Bonni’s group reported that MEF2A sumoylation participates in presynaptic differentiation in the rat brain (Yamada et al., 2013). Indeed, while the *in vivo* knockdown of MEF2A in the rat cerebellar cortex increases the density of orphan presynaptic sites, the sumoylated transcriptional repressor form of MEF2A drives the suppression of these sites via the direct repression of the gene encoding the presynaptic protein Synaptotagmin 1 (Yamada et al., 2013).

Lu et al. (2013) have engineered SENP2 knockout embryos and used *in vivo* SUMO assays to demonstrate that SENP2, but not SENP1, is the MEF2A desumoylating enzyme. They also showed via co-expression of SENP2 and MEF2A with a luciferase reporter gene a SENP2-dependent increase in MEF2A transcriptional activity (Lu et al., 2013) further highlighting the importance of the SUMO process in the transcriptional regulation mediated by MEF2A.

MEF2C, another member of the MEF2 family, is also a sumoylation substrate (Kang et al., 2006). Sumoylation at K391 repressed MEF2C transcriptional activity without altering its DNA-binding properties. Interestingly, phosphorylation at S396 in MEF2C, five residues downstream of the sumoylation site, potentiated MEF2C sumoylation (Kang et al., 2006). The phospho-deficient S396A mutant of MEF2C showed a reduced sumoylation *in vivo* with the concurrent increase in its transcriptional activity further confirming that the regulation of MEF2 activities is controlled by the crosstalk between phosphorylation and sumoylation.

The last member of the MEF2 family to be reported a SUMO substrate is MEF2D. MEF2D sumoylation occurs at the lysine 439 residue (Grégoire and Yang, 2005). The authors showed that the K439 SUMO2/3-ylation of MEF2D strongly decreases its transcriptional activity. In agreement with this, they demonstrated that SENP3 activity is able to increase the transcriptional activity of MEF2D by lowering its sumoylation (Grégoire and Yang, 2005). The same group reported that the kinase Cdk5 promotes MEF2D phosphorylation at the serine 444 residue leading to an increased sumoylation of the protein and consequently to the inhibition of the transcriptional activity of MEF2D (Grégoire et al., 2006). Altogether, these data indicate that the transcriptional activity of MEF2 proteins is tightly regulated through the interplay between several PTMs, e.g., phosphorylation, acetylation and sumoylation, to tightly control the developmental expression of essential target genes involved in brain development and plasticity.

#### FOXP2 (Forkhead Box Protein P2)

FOXP2 belongs to the forkhead box (FOX) family of transcription factors. Disruption of the FOXP2 gene has been implicated in a rare and severe form of autosomal-dominant language and speech disorder (Lai et al., 2001). This disorder was first described in a British family (known as the KE family), in which half of their members struggle to develop coordinated orofacial movements. These patients also express incomprehensive written and spoken language, but they do not show any cognitive impairment. All the affected family members carry the missense arginine to histidine mutation at position 553 (R553H) in FOXP2, which abolishes its DNA binding and consequently fails to repress transcription of many target genes (Lai et al., 2001). FOXP2 is mainly expressed during neuronal differentiation in many brain areas including the cortex, basal ganglia, thalamus and hippocampus (Lai et al., 2003). Importantly, FOXP2 regulates expression of genes that are important for neuronal development and synaptogenesis. For instance, FOXP2 regulates the expression of DISC1 that is involved in neurogenesis, synapse regulation, neuronal outgrowth, migration, differentiation and proliferation (reviewed in Brandon and Sawa, 2011).

Only recently, three independent studies reported that FOXP2 is sumoylated *in vitro* and *in vivo* by all SUMO paralogs predominantly at the lysine 674 residue (Estruch et al., 2016; Meredith et al., 2016; Usui et al., 2016). They further showed that FOXP2 interacts with the E3 SUMO ligases PIAS1 and PIAS3 promoting FOXP2 sumoylation (Estruch et al., 2016; Usui et al., 2016) whereas SENP2 activity significantly decreases its sumoylation (Usui et al., 2016). The FOXP2-PIAS1/3 interaction leads to redistribution of FOXP2 to the nuclear speckles. Interestingly, abolition of the sumoylation site via the K674R mutation did not cause any changes in FOXP2 stability, transcriptional repression or dimerization with the WT sumoylatable form of FOXP2. The subcellular localization of FOXP2 K674R mutant was reported both *in vitro* and *in vivo* to be increased in the cytoplasm and decreased in the nucleus (Usui et al., 2016). Importantly, the human etiological FOXP2 R553H mutation led to a dramatic decrease in the ability of FOXP2 R553H to be sumoylated (Meredith et al., 2016). They further showed that the pathogenic R553H mutation negatively influences the interaction between FOXP2 and the PIAS ligases (Estruch et al., 2016; Meredith et al., 2016).

The cerebellum harbors important motor coordination and speech functions and the expression of FOXP2 in the cerebellum is restricted to Purkinje cells (PC). Usui et al. (2016) have reported that FOXP2 sumoylation is increased during neuronal differentiation in the cerebellum suggesting a key role for sumoylation in cerebellar development. They further showed using mouse neural progenitor cells that overexpression of the WT form of FOXP2 results in long neurites expressing either the immature neuronal marker Tuj1 or the mature neuronal marker MAP2. Interestingly, the overexpression of the SUMO-deficient K674R FOXP2 mutant failed to promote elongation of Tuj1- and MAP2-positive neurites as effectively as the WT FOXP2 indicating that FOXP2 sumoylation is essential to neuronal maturation. *In utero* electroporation to knockdown FOXP2 expression in the cerebellum led to a dramatic reduction in dendritic outgrowth and arborization of PC (Usui et al., 2016). This reduction was rescued by re-expression of the WT sumoylatable form of FOXP2 but not its sumoylation-deficient K674R mutant. Strikingly, the impairments in cerebellum-based motor behaviors such as righting reflex or negative geotaxis observed in FOXP2 knockdown mice were rescued with the expression of the WT form of FOXP2, but not with its sumoylation-deficient K674R mutant confirming the essential role of FOXP2 sumoylation in the developing cerebellum (Usui et al., 2016).

A knock-in mouse model expressing the pathogenic R552H FOXP2 mutation (corresponding to the human FOXP2 R553H mutation) exhibited an immature development of the cerebellum with impaired neuronal migration and autism-related deficits such as decreased ultrasonic vocalizations (Fujita et al., 2008). These vocalization defects were rescued by introducing the WT form of FOXP2 but not its sumoylation-deficient mutant (Usui et al., 2016) further demonstrating that impaired FOXP2 sumoylation could participate in the etiology of FOXP2-related developmental verbal/vocal communication in mammals.

#### CASK (Calcium/Calmodulin-Dependent Serine Protein Kinase)

CASK is a member of the membrane-associated guanylate kinase (MAGUK) protein family. MAGUK proteins have scaffolding properties and interact with many proteins involved in spinogenesis. CASK expression is high in the mammalian brain and extremely critical as its genetic deletion in mice causes neonatal lethality. Mutations within the CASK gene on the X-chromosome have been identified in human patients presenting severe neurological defects, microcephaly and mental impairments, highlighting an essential role of the CASK protein during the brain development (Hsueh, 2009; Hackett et al., 2010).

At the molecular level, CASK binds to a myriad of proteins important for embryonic development, synapse formation and plasticity (Hsueh, 2006). For instance, CASK interacts with the adhesion molecules, e.g., neurexin and syndecans, with cytoplasmic adaptor proteins such as Mint1, SAP97 and CIP98, and with calcium channel proteins. CASK also participates in the regulation of synaptic transmission via its indirect interaction with vesicles that transport the NMDAR subunit NR2B to the plasma membrane (Huang and Hsueh, 2009; Setou et al., 2000).

CASK functions as a multidomain scaffolding protein and has been shown to be sumoylated on the lysine 679 residue (Chao et al., 2008). The sumoylation of CASK reduces the interaction between CASK and the protein 4.1. Mammalian 4.1 proteins are known to act as hubs for cytoskeleton-membrane protein organization and cellular signaling. Notably, protein 4.1 connects spectrin to the actin cytoskeleton and this interaction is crucial for spinogenesis (Huang and Hsueh, 2009). Therefore, in order to evaluate the role of CASK sumoylation in spinogenesis, the authors fused SUMO1 to CASK and overexpressed this chimearic construct in hippocampal neurons. They showed a dramatic impairment in spine number and size (Chao et al., 2008) indicating that CASK sumoylation is essential for spinogenesis. Interestingly, CASK is also expressed presynaptically and future research could therefore shed light on the role of CASK sumoylation in synaptic vesicles (SVs) trafficking and/or neurotransmitter release.

## Presynaptic Sumoylation

The main function of the presynaptic terminal is to orchestrate the release of neurotransmitter from SVs upon neuronal depolarization (reviewed in Südhof and Rizo, 2011; Südhof, 2013). This essential activity-dependent process requires a tightly controlled spatiotemporal regulation of protein-protein interactions between a myriad of molecules to achieve the calcium-dependent fusion of SVs with the presynaptic membrane and the subsequent release of the neurotransmitter in the synaptic cleft. These dynamic events are mostly regulated via PTMs and sumoylation is clearly emerging as a key process at the presynapse.

**Figure 2:**
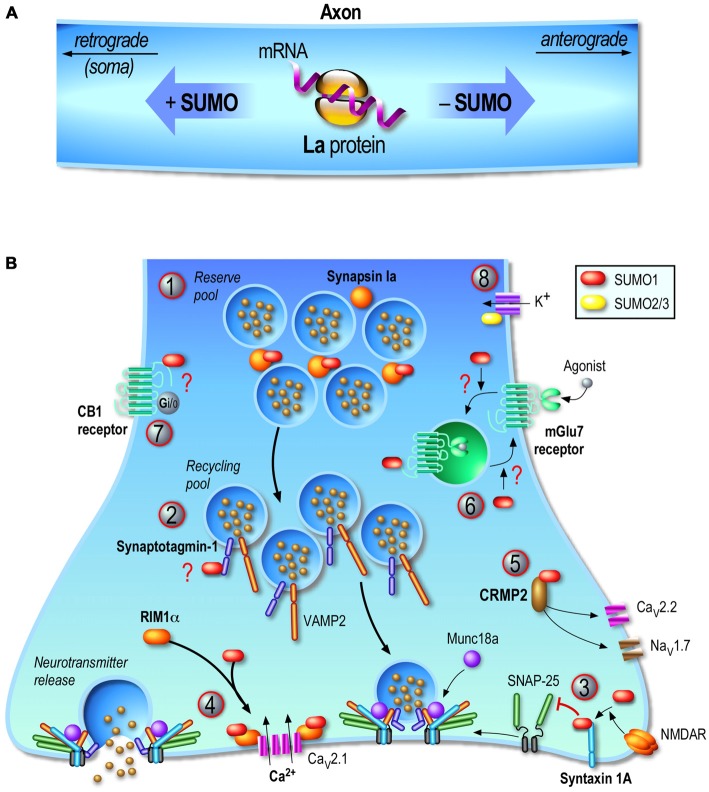
**Regulation of the presynaptic function by sumoylation.** Consistent with the emerging presynaptic functions of sumoylation, its enzymatic machinery is localized at the presynapse and several presynaptic proteins are SUMO subtrates. **(A)** Transport of mRNAs along axons is a key mechanism to dynamically control the function of proteins in growth cones. The axonal mRNA-binding protein **La** is a SUMO substrate. La is transported toward the end of the axon by its association to kinesins while sumoylated La proteins are bound to dyneins and therefore undergo retrograde transport toward the soma. **(B)** Presynaptic sumoylation emerges as a central protein modification acting at several stages of the neurotransmitter release mechanism. **(1)** Sumoylation of **Synapsin Ia** (SynIa) potentiates its association with synaptic vesicles and thus participates in the clustering of these vesicles at the presynapse. **(2)** **Synaptotagmin-1** is sumoylated *in vivo* but the precise function of this modification is still not known. **(3)** **Syntaxin-1A** sumoylation is evoked upon NMDA receptor (NMDAR) activation leading to a decreased binding to SNAP-25 and VAMP-2 and thus acting as a key presynaptic regulator of vesicle endocytosis. **(4)** **RIM1α** sumoylation is required for presynaptic exocytosis since depolarization-evoked vesicle exocytosis with a non-sumoylatable RIM1α mutant is dramatically impaired. This effect is mainly due to a defect in presynaptic calcium entry following neuronal activation since RIM1α sumoylation enables the binding to Cav2.1 calcium channels and coordinates the presynaptic Ca^2+^ entry. **(5)** **CRMP2** is a SUMO substrate and dynamically reduces Ca^2+^ entry through the presynaptic voltage-gated Ca^2+^ channel CaV2.2. CRMP2 sumoylation is also believed to regulate the membrane expression of the sodium channel NaV1.7. **(6)** **mGluR7** is sumoylatable both *in vitro* but also *in vivo* in rat hippocampal and cortical neurons. mGluR7 agonist activation triggers the endocytosis of the WT mGluR7 but not the internalization of its non-sumoylatable mutant suggesting that sumoylation acts on the endocytic pathway. However, overexpressing the desumoylase SENP1 increases the pool of internalized mGluR7, which rather implies that mGluR7 sumoylation is important for recycling of these receptors back to the plasma membrane and not for the receptor endocytosis *per se*. **(7)** Activation of **CB1 receptors** in rat cortical neurons increases the overall SUMO1 conjugation. CB1 receptors are potentially sumoylated in resting cells but not in CB1 receptor-activated conditions. However, the confirmation that these receptors are sumoylated at presynaptic sites and whether the SUMO modification impacts presynaptic endocannabinoid functions are still not determined. **(8)** **Kv potassium channels** play critical roles in neuronal excitability and sumoylation of a number of these channels (Kv1.1, Kv2.1, Kv7.2, Kv7.3) have been reported to act as molecular regulators of their intrinsic activity. Question marks in red indicate that the physiological consequences of the target protein sumoylation are still not clearly defined.

Feligioni et al. (2009) used a modified synaptosomal preparation protocol to trap exogenous conjugatable SUMO1 polypeptides or the catalytically active domain of the desumoylation enzyme SENP1 in synaptosomes to respectively increase or decrease the presynaptic sumoylation levels and measure the impact of sumoylation on glutamate release. They reported that the increase in presynaptic sumoylation reduced Ca^2+^ influx and decreased glutamate release upon KCl depolarization. In contrast, decreasing presynaptic sumoylation by introducing SENP1 into synaptosomes led to an enhanced Ca^2+^ influx and glutamate release in KCl-stimulated conditions (Feligioni et al., 2009). This study was the first to provide evidence for a direct role of the SUMO process at the presynapse via modulation of calcium influx and glutamate release but the molecular pathway and presynaptic proteins targeted by this PTMs were not described at that time. Since then, several key axonal and presynaptic proteins have been reported to be the target of the SUMO system and a better view about the complexity of this process as well as the functional role of sumoylation at the presynapse is now emerging (Figure 2).

### La Protein

The human La protein was originally identified as an auto-antigen in an immune system disorder called Sjogren’s syndrome. Levels of circulating anti-La autoantibodies are used for the diagnosis of this autoimmune syndrome but also in cases of systemic lupus erythematosus and neonatal lupus syndrome. La is the smallest member (46kDa) but the most abundant of the La-related protein (LARP) family (reviewed in Stavraka and Blagden, 2015). Its particular LAM motif adopts a special conformation commonly seen in DNA transcription factors and its RNA-interacting motif RRM allows the binding, protection and axonal transport of many mRNAs. However, how the expression and function of La are regulated remains largely unexplored. To date, it has been shown that phosphorylation of La regulates its activity and possibly its ability to recognize mRNAs. Two kinases, CK2 and Akt, have been so far identified to phosporylate the La protein (Broekhuis et al., 2000; Brenet et al., 2009; Bayfield et al., 2010). Furthermore, (van Niekerk et al., 2007) reported that La is a SUMO substrate and that sumoylated La binds to dynein allowing its retrograde axonal transport. Conversely, the native non-sumoylated La interacts with kinesin and undergoes anterograde axonal transport (Figure 2A). This pioneer study showed that sumoylation is a key regulatory mechanism for transporting mRNAs towards their local translation sites, which represents a crucial process for the maintenance of the axonal and growth cone pool of proteins that are required for synaptic transmission.

### Kv Channels (Voltage-Gated Potassium Ion Channels)

Kv channels form potassium-selective pores that span through the plasma membrane and are essential for the generation of action potentials and the control of neuronal excitability. Mutations in subunits forming some of these channels have been implicated in epilepsies and sudden unexplained death in epilepsy (SUDEP). Investigations into the regulatory roles of sumoylation on potassium channel activities have revealed exciting features. However, most of these works were not achieved in neurons since Kv channels also regulate the excitability of many non-neuronal cells (recently reviewed in Wu et al., 2016). Hereafter, we describe the functional effects of sumoylation of voltage-gated potassium channels in the CNS.

Potassium Kv1.1 channels are abundantly expressed in the brain and localize in large axons where they form tetramers with Kv1.2 subunits. These channels regulate action potential propagation, neuronal firing and neurotransmitter release (Dodson and Forsythe, 2004). Mutations within the human gene encoding Kv1.1 have been associated with partial epilepsy and episodic ataxia in humans (Zuberi et al., 1999). Knock-in mice with Kv1.1 mutations also exhibit hippocampal hyperexcitability, severe epilepsy and premature death (Glasscock et al., 2007). Qi et al. (2014) engineered a post-natal deficient SENP2 mouse model that develops spontaneous seizures and sudden death. They also reported that the SENP2 deficiency results in increased levels of sumoylation for several potassium channels known to impact neuronal excitability including the Kv1.1 that is modified by both SUMO1/2 and colocalizes with SENP2 in hippocampal neurons. However, the sumoylation of Kv1.1 did not significantly affect its channel properties and activity. Interestingly, the authors have also reported in this work that the Kv7.2 is hyper-sumoylated by SUMO2/3 in hippocampal neurons. Kv7 potassium channels play critical roles in neuronal excitability. Two Kv7 members, Kv7.2 and Kv7.3, are highly expressed in neurons and generate the M-current that is important for firing action potentials. Strikingly, the hyper-sumoylation of Kv7.2 resulted in a significant decrease in the depolarizing M-current in SENP2-deficient hippocampal CA3 neurons and consequently led to neuronal hyperexcitability, severe seizures and ultimately, to sudden death of mice by a maximum of 8 weeks of age (Qi et al., 2014). These symptoms were prevented by administration of an approved anti-epileptic drug retigabine. This effective drug acts as a specific Kv7.2 opener and counteracts neuronal hyperexcitability. However, how this drug impacts the sumoylation levels of Kv7.2/7.3 in hippocampal neurons has not been investigated.

Voltage-gated Kv2.1 channels have also been shown to be the target of the SUMO system. While sumoylation of these channels was initially demonstrated in native pancreatic cells where it regulates beta-cell excitability (Dai et al., 2009), Plant et al. (2011) reported a functional role of Kv2.1 sumoylation in hippocampal neurons. Kv2.1 potassium channels are important in neurons for determining the activity-dependent excitability. They reported that sumoylation occurs at the lysine 470 residue and showed that two Kv2.1 subunits have to be modified within a functional Kv2.1 tetramer to produce full SUMO response. Kv2.1 sumoylation led to a 35 mV shift in the half-maximal activation voltage of the functional channel, which resulted in its increased sensitivity to depolarization (Plant et al., 2011). Therefore, sumoylation of Kv2.1 channels provides a way to directly control neuronal excitability.

### Synapsin Ia

Synapsins are synaptic proteins essential for the establishment, clustering and release of presynaptic vesicles (Cesca et al., 2010). Synapsin Ia (SynIa) is involved in maintaining the reverse pool of synaptic vesicles that is required when neuronal stimulation lasts for longer period of time. Tang et al. (2015) demonstrated that SynIa is sumoylated at the K687 residue and this sumoylation potentiates its association with synaptic vesicles participating in the clustering and anchoring of these vesicles into the presynaptic element (Figure 2B). The lysine-687 to arginine mutation resulted in complete absence of SynIa sumoylation, decrease in the number of releasable synaptic vesicles and impaired exocytosis (Tang et al., 2015). Notably, the A548T mutation in SynIa that co-segregates with autism also impairs SynIa sumoylation. Defects in SynIa sumoylation may therefore be involved in the pathophysiology of neurological disorders through a SUMO-dependent deregulation of SynIa function at the presynapse. Altogether, sumoylation of SynIa appears to be critical for the activity-dependent release of neurotransmitter and may therefore actively participate in synaptic transmission and potentially in long-term synaptic plasticity events.

### Syntaxin-1A

The activity-dependent exocytosis of neurotransmitters at presynaptic sites and the subsequent recycling of synaptic vesicles are essential processes underlying synaptic communication. The exocytotic event is mediated through the action of the SNARE (Soluble N-ethylmaleimide sensitive factor Attachment protein REceptor) protein complex that includes the 35 kDa-membrane protein Syntaxin-1A (Stx1A), SNAP-25 and VAMP-2, and additional proteins such as Munc18, Synaptotagmins and RIM1α (Figure 2B). Stx1A has been reported to be important in neuronal survival (Kofuji et al., 2014), neurotransmitter release and recycling of SV (Watanabe et al., 2013). The role of Stx1A in neurotransmitter release is also supported by studies reporting a possible involvement of Stx1A in the pathophysiology of autism with Stx1A mRNA expression levels being significantly higher in autistic patients compared to controls (Nakamura et al., 2008). Furthermore, the STX1A gene, which is located at the chromosome 7q11.23, has been found duplicated in patients with speech delay and autism spectrum behaviors (Berg et al., 2007; Depienne et al., 2007). All these findings therefore converge to the idea that Stx1A is critically important for synapse formation, presynaptic function and neuronal transmission in the developing brain.

Interestingly, Stx1A has been recently reported as a novel presynaptic sumoylation target (Craig et al., 2015). Stx1A sumoylation is evoked upon NMDAR activation or following KCl-depolarization in hippocampal neurons. This activity-dependent sumoylation occurs at three lysine sites (K252, 253, 256) and reduces Stx1A binding to SNAP-25 and VAMP-2, but not to Munc18a. Importantly, neuronal expression of a non-sumoylatable form of Stx1A via the mutation of the three SUMO sites into arginine residues, leads to a significant increase in presynaptic vesicle endocytosis (Craig et al., 2015). This suggests that Stx1A sumoylation is critically involved in the maintenance of the balance between SV endocytosis/exocytosis and subsequently in neurotransmitter release. However, how exactly the sumoylated form of Stx1A enhances SV endocytosis as well as how Stx1A desumoylation occurs in this context has not yet been investigated.

### Synaptotagmin-1

Membrane fusion is a key mechanism occurring for many processes including protein/lipid transport, hormone and neurotransmitter release. Membrane fusion at presynaptic site involves not only the SNARE proteins but also several other presynaptic factors to orchestrate neurotransmission in a timely dependent way (reviewed in Südhof and Rizo, 2011; Südhof, 2013). Among these are calcium sensor proteins called synaptotagmins. To date, 16 isoforms of synaptotagmins have been identified in mammals that either colocalize with synaptic/secretory vesicles or are distributed at the plasma membrane. Although not directly related to presynaptic exocytotic function, Dai et al. (2011) reported that SENP1 overexpression enhances insulin exocytosis in pancreatic β-cells via the association of SUMO1 to Synaptotagmin VII. More interesting is the presynaptic function of Synaptotagmin-1 (Syt1) sumoylation. Syt1 is well known to exert important roles at the presynapse to sense the calcium influx that arises through the activated voltage-gated calcium channels and thus Syt1 participates in neurotransmitter release (Figure 2B).

To assess the role of sumoylation in neuronal function, the Fraser lab used a proteomic approach on transgenic mice that exclusively over-expressed the human form of SUMO1 in neurons (Matsuzaki et al., 2015). The effect of this over-expression was a simultaneous increase in the level of non-conjugated SUMO1 proteins and in the proportion of high molecular weight SUMO1-modified targets in transgenic brains compared to WT brains. The levels of protein expression of the SUMO enzymes as well as the free fraction of SUMO2/3 proteins in transgenic brain remained similar to those measured in WT animals. Using mass spectrometry, the authors confirmed that many of these SUMO1 targets were neuron and synapse-specific. Importantly, the authors described the sumoylation of Syt1 and showed that Syt1 sumoylation was upregulated in these transgenic mice (Matsuzaki et al., 2015). Using field potential recording in acute hippocampal slices from SUMO1-transgenic brains, they reported a deficit in basal transmission suggesting a decrease in synaptic activity and/or a loss of functional synapses. They also showed that a form of short-term synaptic plasticity dependent on presynaptic mechanisms, named paired pulse facilitation, is impaired in SUMO1-transgenic brain slices, which suggests that SUMO1 over-expression leads to defects in functional presynaptic mechanisms (Matsuzaki et al., 2015). They further showed that SUMO1-over-expressing hippocampal cells exhibit a dramatic loss of dendritic spines that leads to impairment in contextual fear memory (Matsuzaki et al., 2015). While the over-expression of SUMO1 in neurons leads to multiple alterations, the functional and physiological functions of Syt1 sumoylation are yet to be described. Clearly, the hyper-sumoylation observed for Syt1 in SUMO1-transgenic mice cannot be taken as the unique cause to explain all the physiological deficits reported in these animals. However, this work confirmed the importance of a controlled equilibrium between sumoylation and desumoylation since a small and uncompensated increase in neuronal sumoylation directly impacts synaptic architecture, cell communication and memory formation.

### RIM1α (Rab3-Interacting Molecule 1α)

Among the proteins of the presynaptic active zone that have been extensively studied are the RIM protein family. RIMs interact either directly or indirectly with several presynaptic proteins including Rab3a, synapsin-1, Syt1A, Munc13–1, and the voltage-gated Ca^2+^ channels (Calakos et al., 2004). These scaffolding proteins are crucial to the active zone function and consequently to synaptic transmission (Figure 2B). Specifically, RIM1α has been implicated in the docking/priming of synaptic vesicles but also in short and long-term synaptic plasticity (Castillo et al., 2002; Dulubova et al., 2005). It is now generally believed that RIM1α plays key roles in diverse presynaptic functions, however, the regulatory mechanisms at the presynaptic site have not been fully elucidated. A recent study from the Henley lab reported that RIM1α is a SUMO substrate (Girach et al., 2013). They showed that RIM1α sumoylation occurs only on the lysine 502 residue independently of the neuronal activity. Using molecular replacement experiments, they have substituted the endogenous RIM1α in hippocampal neurons by the non-sumoylatable RIM1α-K502R mutant. While the presynaptic localization of both the WT and non-sumoylatable exogenous RIM1α remained unchanged, there was a marked decrease in the depolarization-evoked SV exocytosis with the K502R mutant indicating that RIM1α sumoylation is required for presynaptic exocytosis (Girach et al., 2013). They further demonstrated that the outcome measurements of the mutant were due to a defect in calcium entry following depolarization since RIM1α sumoylation enables the clustering of Cav2.1 calcium channels. Altogether, (Girach et al., 2013) uncovered an additional important presynaptic function for the SUMO process. As there are other isoforms of RIM proteins that are involved in modulation of presynaptic functions, it would be of interest to investigate whether and how sumoylation can impact on these proteins.

### CRMP2 (Collapsin Response Mediator Protein 2)

CRMP2 is a microtubule-binding protein that was originally identified for its roles in regulation of axonal guidance in neuronal polarity and more recently, in presynaptic functions including axonal transport and neurotransmitter release (for a recent review on CRMP2 see Ip et al., 2014). CRMP2 dynamically interacts with the presynaptic N-type voltage-gated Ca^2+^ channel (CaV2.2) and disruption of this complex reduces pain in a rodent model of neuropathic pain. Thus, investigation into CRMP2 mechanisms of action is of interest to understand its role in pain and identify potential therapeutic targets (Brittain et al., 2011). CRMP2 has been reported to be sumoylated *in vitro* on the lysine 374 residue and preventing CRMP2 sumoylation did not impair its ability to promote neurite outgrowth (Ju et al., 2013). Using calcium imaging on primary rat cultures of dorsal root ganglion (DRG) neurons, the authors showed that the non-sumoylated form of CRMP2 differentially affects the calcium influx in depolarized DRGs when compared to WT CRMP2 expression suggesting that CRMP2 sumoylation acts as a negative modulator of presynaptic calcium influx.

The same group later confirmed that both the WT and the SUMO-deficient CRMP2 are robustly expressed in catecholaminergic cells (CAD) and are able to promote neurite outgrowth in rat DRG neurons (Dustrude et al., 2013). They have also reported that the sodium channel NaV1.7 is regulated by CRMP2 sumoylation. Preventing sumoylation by over-expressing SENP1 and SENP2 enzymes in WT CRMP2-expressing CAD cells decreased the NaV1.7 currents. Accordingly, there was a significant decrease in the levels of surface-expressed NaV1.7 in CAD cells expressing the SUMO-deficient form of CRMP2. NaV1.7 currents were also decreased in sensory neurons expressing the non-sumoylatable CRMP2 K374A mutant (Dustrude et al., 2013).

Overall these two reports highlight the putative function of CRMP2 sumoylation in the regulation of calcium and sodium channels; however, the authors did not demonstrate CRMP2 sumoylation *in vivo*. It is also to be determined whether CRMP2 sumoylation directly modifies the activity or the surface expression of the two channels. Further work will therefore be required to clarify the functional role of CRMP2 sumoylation at presynaptic sites.

### Metabotropic Glutamate Receptors

Metabotropic glutamate receptors (mGluRs) form a family of G-protein coupled receptors that are centrally involved in excitatory neurotransmission and synaptic plasticity. mGluRs are divided into three groups based on their sequence homology, G-protein coupling and ligand specificity (reviewed in Niswender and Conn, 2010). The group III consists of mGluR4, 6, 7 and 8, and is of particular interest since these receptors typically exert presynaptic inhibitory functions. In the past years, several group III mGluRs have been shown to be sumoylated mainly *in vitro* but also *in vivo*, however until recently, there was no compelling evidence regarding the functional roles for such modifications (Tang et al., 2005; Wilkinson et al., 2008; Dütting et al., 2011; Wilkinson and Henley, 2011).

The functional role of sumoylation in mGluRs has been so far addressed solely for the mGluR7. These receptors are widely expressed presynaptically and modulate excitatory neurotransmission as well as synaptic plasticity by inhibiting neurotransmitter release (reviewed in Niswender and Conn, 2010). C-terminal truncated forms of mGluR7 were found to be sumoylated at the K889 residue *in vitro* (Wilkinson et al., 2008; Wilkinson and Henley, 2011). In a recent study, Choi et al. (2016) confirmed that mGluR7 is a SUMO substrate *in vitro*. They have also shown that these receptors are sumoylated *in vivo* in both the rat hippocampus and primary cortical neurons with the mGluR7-K889 residue identified as the sole sumoylation site. While mGluR7 can be sumoylated by both SUMO1 and SUMO2/3 in HEK293T cells, only SUMO1 conjugation was reported in hippocampal homogenates (Choi et al., 2016). Since the sumoylation process has been directly involved in the endocytosis of glutamate receptors in hippocampal neurons (Martin et al., 2007a), the authors investigated whether sumoylation has an effect on mGluR7 internalization (Figure 2B). Constitutive agonist-independent endocytosis of the non-sumoylatable mGluR7 K889R mutant was increased compared to the WT control receptor. Addition of L-AP4 mGluR7 agonist to the cells expressing WT receptors triggers the endocytosis of mGluR7. This increase in agonist-evoked mGluR7 endocytosis was not seen for the non-sumoylatable mGluR7. The authors attributed this lack of effect to the sumoylation process directly acting on the endocytic pathway. However, they cannot rule out that sumoylation rather impact on the recycling properties of the pathway. It is indeed likely that sumoylation acts after the endocytosis of mGluR7 by preventing the recycling of the non-sumoylatable receptor. This is in line with their data showing that overexpression of SENP1, which prevents sumoylation, leads to an increase in the internalized population of WT mGluR7 similar to the values measured for the endocytosed population of the non-sumoylatable mutant in the absence of SENP1. This could be explained by a decrease in the SUMO-dependent recycling of internalized mGluR7 to the plasma membrane that leads to an increased intracellular pool of receptors. Since this pathway was not assessed, it is difficult to conclude about the exact role of mGluR7 sumoylation in the internalization/recycling process. Furthermore, mGluR7s are primarily expressed at presynaptic sites (Niswender and Conn, 2010). Since the current work (Choi et al., 2016) examined the postsynaptic endocytic properties of an over-expressed tagged version mGluR7, it implies that further work will now be necessary to assess the functional impact of mGluR7 sumoylation at presynaptic sites and whether this SUMO modification influences neuronal excitability and/or synaptic transmission and plasticity.

### Cannabinoid Receptor 1

The endocannabinoid system fulfils complex neuromodulatory functions in brain development and synaptic plasticity (reviewed in Lu and Mackie, 2016). It is composed of endogenous cannabinoid substrates (endocannabinoids), receptors and enzymes that synthesize and degrade endocannabinoids. Strikingly, impairments of the endocannabinoid system have been implicated in several psychiatric disorders. The most abundant endocannabinoid receptors, CB1 and CB2, belong to the family of G-protein coupled receptors, which primarily couple to G proteins of the Gi and Go classes. Their activation leads to inhibition of adenylyl cyclases and modulation of presynaptic voltage-dependent calcium channels as well as certain potassium channels (Lu and Mackie, 2016). CB1 and CB2 receptors are involved in a number of physiological functions, such as gene transcription, cell motility and synaptic communication. CB1 receptors are highly expressed in the cortex, basal ganglia, hippocampus, and the cerebellum. CB1 receptors are primarily present at presynaptic terminals (Figure 2B) while CB2 receptors, which are expressed at a much lower level in the CNS, are mainly expressed in microglia and vascular elements. Activation of CB1 receptors in rat cortical neurons leads to an increase in the overall SUMO1 conjugation as well as an increase in the levels of free SUMO1 (Gowran et al., 2009). The authors further showed that CB1 receptors were potentially sumoylated in basal but not in CB1 receptor-activated conditions (Gowran et al., 2009). However, there have been no reports so far regarding which CB1 receptor residues are sumoylated and whether the SUMO modification regulates presynaptic endocannabinoid functions.

In recent years, a lot of work has been achieved regarding the identification of presynaptic SUMO target proteins and the function of sumoylation at the presynapse, placing the SUMO pathway as a key regulator of protein-protein interactions within this highly crowded environment. Despite these efforts it is still unknown how is this timely dependent sumoylation/desumoylation process orchestrated and future work will be required to decipher how the targeting, the trafficking and the activity of the sumoylation and desumoylation enzymes are regulated in an activity-dependent manner at presynaptic sites.

## Postsynaptic Sumoylation

Spines are small protrusions on dendritic membranes receiving inputs from axonal termini. Dendritic spines represent the postsynaptic elements that consist in a head connected to the dendritic shaft by a narrow neck and contain multiple synaptic actors, which interact in a coordinated manner to allow synaptic communication. The first demonstration that sumoylation acts directly within the synapse has been provided in 2007 with the immunodetection of many unidentified sumoylated substrates in rat hippocampal PSD95-positive synaptic fractions as well as with the immunolocalization of the sole SUMO conjugating enzyme Ubc9 at postsynaptic sites (Martin et al., 2007a). This work has also identified and characterized the first synaptic sumoylated substrate i.e., the kainate receptor (KAR) subunit GluK2, and therefore has opened new avenues for investigation of the sumoylation process in the brain (Martin et al., 2007a).

### Kainate Receptors

Kainate receptors are ionotropic glutamate receptors that are functionally active as tetramers composed of the subunits GluK1–5 (formerly named GluR5–7, KA1 and KA2). KARs play important roles for synaptic transmission as well as neuronal excitability (Contractor et al., 2011). They are expressed at many synapses both pre- and postsynaptically but also extrasynaptically, where they regulate neuronal excitability. At the presynapse, they participate in the release of neurotransmitters, whereas postsynaptically they contribute to synaptic transmission. The GluK2 subunit directly interacts with the conjugating enzyme Ubc9 and is a sumoylation substrate in rat hippocampal neurons (Martin et al., 2007a). GluK2 sumoylation by SUMO1 occurs in an activity-dependent manner on its C-terminal domain at the single lysine 886 residue. Since this report, several additional studies have confirmed the SUMO state of GluK2 in neurons (Konopacki et al., 2011; Zhu et al., 2012; Choi et al., 2016). Agonist activation causes the endocytosis of GluK2 receptors via a PKC-dependent pathway (Martin and Henley, 2004). Interestingly, the binding of glutamate or kainate to GluK2 leads to its sumoylation at the plasma membrane and represents a trigger for the activated receptors to be internalized. Interestingly, postsynaptic KAR responses at hippocampal mossy fiber-CA3 synapses decrease when postsynaptic sumoylation is promoted by infusing SUMO1 postsynaptically and conversely, postsynaptic responses largely increase in desumoylation conditions using infusion of the catalytic domain of SENP1 (Martin et al., 2007a). Consistent with earlier publication (Martin and Henley, 2004), PKC activation has been shown to be essential to GluK2 internalization (Konopacki et al., 2011; Chamberlain et al., 2012). PKC phosphorylation at the serine 868 in GluK2 is a prerequisite for its sumoylation and subsequent endocytosis (Konopacki et al., 2011; Chamberlain et al., 2012).

### Arc (Activity-Regulated Cytoskeleton-Associated Protein/Activity-Regulated Gene 3.1)

The immediate early gene Arc is capable of coupling changes in neuronal activity to synaptic plasticity events in a tightly regulated way (reviewed in Bramham et al., 2010). Arc is a unique gene required for consolidation of synaptic plasticity and LTP. Transcription of Arc gene is strongly induced by synaptic activity. Arc mRNAs are rapidly transported into dendrites where they undergo local translation at synaptic sites. Therefore it is not surprising that Arc exhibits key roles in protein synthesis-dependent forms of synaptic plasticity and in consolidating different forms of memory (Bramham et al., 2010). Interestingly, Arc levels are also controlled by ubiquitination and proteasomal degradation as it was shown that defective Arc ubiquitination increases Arc levels leading to the concurrent decrease in synaptic AMPAR receptors (Greer et al., 2010).

AMPAR are heterotetrameric (GluA1-A4) glutamate-gated ion channels that underpin the vast majority of fast excitatory glutamate neurotransmission in the CNS. Interestingly, a chronic abolishment of neuronal activity promotes AMPARs membrane expression and in contrast an increase in neuronal activity leads to decreased surface AMPARs. However, the mechanisms, by which the number and composition of AMPARs change, are still not fully understood. To date, it is believed that Arc participates in the internalization of AMPAR from the plasma membrane through its interaction with the endocytic endophilin-3 and dynamin-2 proteins (Chowdhury et al., 2006).

The Henley group reported that Arc is a sumoylation substrate with the lysine 110 and 268 residues being the sites of sumoylation (Craig et al., 2012). They also showed that the suppression of network activity with the sodium channel blocker tetrodotoxin (TTX) induces SENP1 degradation leading to the concurrent increase in SUMO1- and SUMO2/3-modified protein levels in rat cortical neurons in primary culture (Craig et al., 2012). The level of Arc proteins was dramatically reduced in TTX conditions independently of its sumoylatable ability indicating that sumoylation does not exert any stabilizing effect on Arc. The prolonged exposure to TTX also directly increases the membrane expression of GluA1 subunits of AMPARs, a process named synaptic scaling (Turrigiano, 2008). This effect in surface-expressed AMPAR in TTX condition was prevented when the catalytic domain of SENP1 was expressed, revealing the involvement of the SUMO process in this homeostatic scaling effect (Craig et al., 2012). However, how the SUMO process participates in the regulation of AMPAR levels at the plasma membrane, how Arc sumoylation levels are modulated by the TTX treatment and how Arc sumoylation impacts on AMPAR trafficking still remain open questions.

### Regulation of the Sumoylation Pathway at the Postsynapse

**Figure 3:**
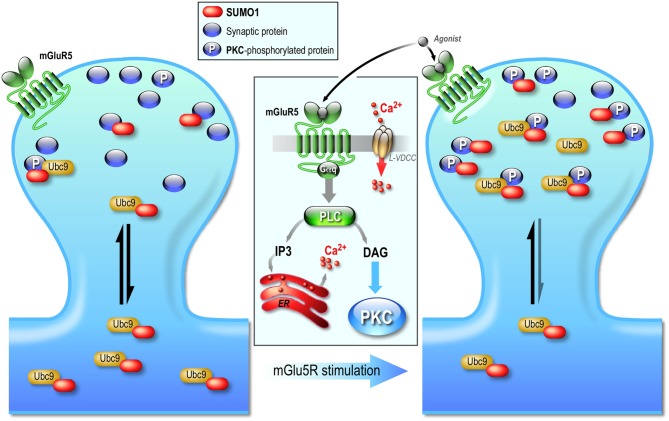
**Postsynaptic regulation of the SUMO pathway.** The diffusion of Ubc9, the sole conjugating enzyme of the SUMO system is regulated by synaptic activity. Neuronal activation increases the residency time of the SUMO-conjugationg enzyme Ubc9 at the post-synapse. This synaptic regulation is independent of NMDAR activation but involves an mGlu5R-dependent signaling pathway that leads to PKC activation. The effect of this activity-dependent PKC phosphorylation is the increased anchoring of Ubc9 to synaptic phosphorylated protein substrates, which ultimately enhances sumoylation at synapses. Activation of mGlu5R therefore promotes a transient diffusional trapping of Ubc9 in activated spines via a PKC-dependent pathway, which in turn contributes in the regulation of neuronal excitability.

Despite numerous publications demonstrating the postsynaptic involvement of sumoylation, it is only recently that some of the mechanisms regulating this post-translational system at the post synapse were reported (Loriol et al., 2014; Figure 3). Indeed, using a combination of pharmacological tools with synaptic biochemistry and restricted photobleaching/photoconversion of individual hippocampal spines, our group demonstrated that the synaptic diffusion of Ubc9, the sole conjugating enzyme of the sumoylation pathway, is regulated by synaptic activity on a rapid timescale. The synapto-dendritic diffusion of Ubc9 remained unchanged upon the activation of NMDARs but was altered through the activation of group I metabotropic mGluR5 receptors (see Niswender and Conn, 2010 for a comprehensive review on mGluRs signaling pathways). Increasing synaptic activity with a GABA_A_ receptor antagonist or directly activating mGlu5R increases the synaptic residency time of Ubc9 in a PKA-independent but PKC-dependent manner. This transient synaptic diffusional trapping of Ubc9 enhanced its recognition to synaptic PKC-phosphorylated substrates and consequently leads to the increase in synaptic sumoylation (Loriol et al., 2014; Figure 3). However, despite this first demonstration that the sumoylation pathway is activity-dependently regulated at postsynaptic sites, future work will now be required to identify the nature of these synaptic mGlu5R-activated SUMO substrates to further decipher the synaptic functions of sumoylation.

Altogether, the data from the above sections clearly establish that the sumoylation machinery is partly targeted to, localized and regulated at pre- and postsynaptic sites to modulate in an activity-dependent manner the levels of synaptic sumoylation and in turn, the synaptic function. Furthermore, a growing number of SUMO substrates were recently identified in axons, dendrites and synapses and shown to fulfil essential physiological functions on synaptic communication and plasticity (Shalizi et al., 2006, 2007; Martin et al., 2007a; Chao et al., 2008; Konopacki et al., 2011; Chamberlain et al., 2012; Craig et al., 2012, 2015; Girach et al., 2013; Jaafari et al., 2013; Loriol et al., 2013, 2014; Tang et al., 2015; Tai et al., 2016) revealing the sumoylation process as an essential modulator of the synaptic function. Strikingly however, a study combining the use of a double-tagged His-HA-SUMO1 knock-in mouse model and mass spectrometry analysis failed to detect any synaptic SUMO substrates nor any colocalization between His-HA-SUMO1 at synapses (Tirard et al., 2012). The explanation for these rather stark differences is still unclear but the authors demonstrated that the levels of SUMO conjugation decreased in the knock-in model compared to WT animal suggesting that the dual SUMO tag partly impairs the sumoylation process. The direct outcome of this observation is that the synaptic sumoylation levels may become too low and below the detection sensitivity of their analysis method. Despite these data, an increasing number of exciting studies from independent groups worldwide including ours is now available demonstrating that sumoylation takes place in neurons and at synapses to regulate synaptic communication.

## Sumoylation in Synaptic Plasticity

Synaptic plasticity is characterized by the ability of a synapse to change in strength over long periods of time and this process is now widely accepted as the cellular model of learning and memory. Sumoylation, as described above, is centrally involved in the fine-tuning of neuronal excitability and the regulation of several pre- and postsynaptic proteins important for synaptic transmission. In recent years, several pieces of evidence have accumulated implicating the sumoylation process in plasticity events.

Phosphorylation of the cAMP-responsive element binding protein (CREB) at the serine 133 residue via different signaling cascades, e.g., Ras/ERK, Akt kinase, calcium/calmoduin-dependent kinases II and IV, protein kinase A, regulates memory formation and neuronal survival during development and leads to transcription of genes required for activity-dependent brain plasticity, which makes CREB a prototypic transcriptional factor of cognitive function of the brain (Cohen and Greenberg, 2008; Bell et al., 2013). In an in-depth study published recently, Chen et al. (2014) investigated the role of CREB sumoylation and its interplay with phosphorylation in the rat hippocampal CA1 region. They showed that CREB sumoylation is enhanced in the presence of PIAS1, and NMDA injection in the CA1 region increases CREB sumoylation. Moreover, the spatial training in rats increases CREB phosphorylation after 1 day of training. After 2 and 5 days the phospho-CREB levels remained unchanged compared to untrained control animals, whereas CREB sumoylation increased significantly suggesting a molecular regulatory switch between phosphorylation and sumoylation during this learning process. In addition, CREB sumoylation enhanced the transcription of growth factor Brain-Derived Neurotrophic Factor (BDNF). Transduction of CREB-SUMO1 fusion vector to the rat CA1 region increased spatial learning and memory, whereas PIAS1 knock-down decreased CREB sumoylation and impaired spatial learning and memory (Chen et al., 2014). Importantly, the authors provided evidence that preventing CREB phosphorylation completely abolishes CREB sumoylation, however preventing CREB sumoylation on two most prominent sumoylation sites increases CREB phosphorylation in the CA1 region. Clearly, there is a regulatory interplay between these two modifications and it will be of interest to examine whether deregulation of the CREB phosphorylation/sumoylation crosstalk is relevant in cognitive disorders.

### LTP (Long-Term Potentiation)

LTP is characterized by a long-lasting increase in synaptic strength that involves in most cases an activity-dependent increase in the functionality and the number of postsynaptic AMPAR (Kneussel and Hausrat, 2016).

Jaafari et al. (2013) applied a chemically-induced LTP assay (Chem-LTP) on cultured rat hippocampal neurons to investigate the role of sumoylation in AMPARs surface expression. This pharmacological approach was previously reported to significantly increase the surface level of AMPARs (Lu et al., 2001). Chem-LTP led to an increase in dendritic and synaptic SUMO1 immunoreactivity as well as a large increase in Ubc9 and SUMO1 mRNAs in soma and dendrites. Interestingly, the over-expression of a catalytically active domain of the desumoylase SENP1, but not its catalytically inactive mutant, prevented the increase in SUMO1 mRNA and in surface expressed AMPAR upon Chem-LTP (Jaafari et al., 2013). These results are in favor of an active role of the sumoylation process in the control of AMPAR surface expression during LTP. However, the precise mechanism by which the SUMO process acts on AMPAR surface expression is still not clear.

The prion-like Cytoplasmic Polyadenylation Element-Binding protein 3 (CPEB3) regulates the translation of several mRNAs involved in synaptic plasticity (Pavlopoulos et al., 2011; Fioriti et al., 2015). Previous studies reported that CPEB exists as a soluble inactive or insoluble aggregate-prone active protein (Si et al., 2010), both of which localize at the synapse (Drisaldi et al., 2015). When aggregated, active CPEB3 can initiate the translation of specific target mRNAs such as those coding for the AMPAR subunits GluA1 and GluA2 (Pavlopoulos et al., 2011; Fioriti et al., 2015). Interestingly, sumoylation of CPEB3 by SUMO2 was shown to regulate its oligomerization capacity and neuronal activity-dependent translation of target mRNAs (Drisaldi et al., 2015). In basal state CPEB3 is sumoylated and acts as a translation repressor. *In vitro* and *in vivo* stimulation of hippocampal neurons triggered CPEB3 desumoylation leading to its aggregation and mRNA translation. The authors found that the uncleavable SUMO2-CPEB3 construct is soluble compared to the non-sumoylated CPEB3 and showed a decreased ability to aggregate leading to inhibition of mRNA translation. Stimulation of hippocampal neurons with glycine led to an increase in the number of filopodia (immature spines), which was not observed when neurons expressed SUMO2-CPEB3 (Drisaldi et al., 2015). Importantly, CPEB3 was reported to induce SUMO2 mRNA translation upon glycine stimulation. These data suggest that the SUMO process operates as a regulatory loop influencing the translation activity of CPEB3, which in turn modulates the levels of SUMO2 mRNA.

The sumoylation process is required for the expression of LTP (Lee et al., 2014). Indeed, by combining the use of WT or catalytically inactive forms of the cell permeable TAT-Ubc9 and LTP protocols in acute CA1 hippocampal slices, the authors showed that LTP is significantly reduced when sumoylation is prevented by the dominant negative Ubc9 mutant (Lee et al., 2014). This LTP inhibition was observed without any impact on basal transmission. Lee et al. (2014) confirmed their initial results using the catalytic domain of the desumoylase SENP1 in the patch pipette as used previously (Martin et al., 2007a). They showed that inclusion of the active SENP1, but not its catalytically inactive mutant, fully blocked the induction of LTP in CA1 pyramidal neurons confirming that the SUMO pathway is involved in the expression of long-term plasticity events (Lee et al., 2014). They subsequently demonstrated that infusion of the dominant negative form of TAT-Ubc9 *in vivo* impairs the hippocampal-dependent learning and memory (Lee et al., 2014).

More recently, several MeCP2 gene mutations in patients with Rett syndrome patients were shown to decrease MeCP2 sumoylation (Tai et al., 2016). The authors also demonstrated that the re-expression of the WT form of MeCP2 in CA1 hippocampal neurons rescued the deficits of social interaction and the CA1-LTP impairment observed in MeCP2 conditional knockout mice. Interestingly, re-expression of the non-sumoylatable K412R form of MeCP2 in these conditional knockout mice was not able to rescue the LTP in CA1 hippocampal neurons with measured values similar to those obtained in MeCP2 KO animals (Tai et al., 2016). Altogether, these data reveal a crucial role of MeCP2 sumoylation in social interaction and synaptic plasticity, and suggest that erratic MeCP2 sumoylation may directly participate in the etiology of Rett syndrome.

### LTD (Long-Term Depression)

LTD is a ubiquitous form of activity-dependent long-lasting reduction of synaptic strength characterized by a decrease in the surface expression of neurotransmitter receptors that often results from the remodeling of their intracellular protein-interacting partners via PTMs.

As depicted above, the agonist-dependent sumoylation of the GluK2 subunit at the lysine 886 leads to the internalization of the sumoylated KAR complexes (Martin et al., 2007a). This process requires a PKC-phosphorylation of the GluK2 C-terminus at the serine 868 residue prior to its sumoylation (Konopacki et al., 2011). Interestingly, it was also reported that both the PKC-phosphorylation of the serine 868 and the subsequent sumoylation are required for the internalization of KARs that occurs during LTD of KAR-mediated synaptic transmission at rat hippocampal mossy fiber synapses (Chamberlain et al., 2012). Thus, this work revealed that the interplay between phosphorylation and sumoylation of GluK2 is important for activity-dependent KAR synaptic plasticity.

## Sumoylation in Synaptopathies

The human synaptic proteome is composed of hundreds of different proteins in many copies and mutations in the encoding genes lead to more than hundred brain disorders (reviewed in van Spronsen and Hoogenraad, 2010; Grant, 2012). Spine architecture, synaptic proteome and neuronal functions are strongly correlated features, which is never more apparent than in pathological conditions. Notably, synaptopathies that are characterized by alterations in spine morphology, synapse number and synaptic function are increasingly seen as central feature in major psychiatric, brain developmental and neurodegenerative diseases. These diseases constitute a major social and economic burden in our societies and it is therefore essential to gain a better insight into the underlying molecular and cellular mechanisms prior to developing effective diagnostic, preventative and eventually therapeutic strategies.

Since the sumoylation pathway is emerging as a critical regulator of neuronal and synaptic function under normal conditions, it is not surprising to see more and more publications reporting defective sumoylation events in wide range of brain disorders. In this section, we review the current knowledge regarding the multiple sumoylation anomalies reported in synaptopathies (Table 1).

**Table 1:** **Sumoylation in synaptopathies**.

Synaptopathy	Implicated SUMO targets and machinery	Effects	Reference
**Down syndrome, Trisomy 21**	*SUMO3*	SUMO3 gene is localized on Hsa21.	Gardiner (2006)
		SUMO3 overdose leads to imbalanced/deregulated sumoylation.
**Parkinson’s disease**	*α-Synuclein*	Sumoylated by SUMO1 and SUMO2/3. Involved in protein aggregation.	Kim et al. (2011), Krumova et al. (2011), and Kunadt et al. (2015)
		Another pathogenic mechanism could include inter-neuronal spreading of **α**-Syn.
	*DJ-1*	PD mutation disrupts DJ-1 sumoylation and decreases its solubility.	Shinbo et al. (2006)
	*Parkin*	Increase in its E3 Ubiquitin ligase activity by non-covalent SUMO1 modification.	Um and Chung (2006)
		Parkin also associates with and targets the SUMO E3 ligase RanBP2 for degradation. Direct implication with PD is still lacking.
**Huntington’s disease**	*Huntingtin*	Sumoylation may act as a prevention mechanism for huntingtin accumulation.	Steffan et al. (2004) and O’Rourke et al. (2013)
**Alzheimer’s disease**	*SAE2, Ubc9, SENP3*	Single Nucleotide Polymorphisms in these genes co-segregate with AD.	Grupe et al. (2007); Weeraratna et al. (2007); Ahn et al. (2009); and Corneveaux et al. (2010)
	*Aβ*	Unclear results about whether sumoylation of A**β** enhances or decreases its aggregation.	Li et al. (2003); Dorval et al. (2007); and Zhang and Sarge (2008)
	*Tau*	Proportion between sumoylated and ubiquitinylated Tau can regulate its degradation/accumulation.	Dorval and Fraser (2006) and Luo et al. (2014)
		Hyper-phosphorylated Tau is immunoreactive for SUMO1.

### Down Syndrome (DS)

The DS or trisomy 21, is caused by an extra copy of all or parts of the long arm of the human chromosome 21 (Hsa21) and is the most common chromosomal abnormality with about 1:1000 births worldwide (Loane et al., 2013). Clinical features are multiple with mild to severe intellectual disabilities, learning defects in short- and long-term memory formation, typical craniofacial appearance, hypotonia and premature aging (Perluigi and Butterfield, 2012). On the neuroanatomical side, DS patients show reduction in brain size and weight, as well as a decrease in neuronal density associated with synaptic abnormalities (Kaufmann and Moser, 2000).

DS is believed to result from a gene dosage imbalance leading to the increased expression of normal chromosome 21 genes. Accordingly, the overexpression of specific genes located in the long arm of Hsa21, such as DS Critical Region 1 (DSCR1), the Amyloid-beta Precursor Protein (APP) and the dual-specificity tyrosine (Y)-phosphorylation regulated kinase 1A (DYRK1A) genes have been reported in DS patients (Antonarakis et al., 2004; Shukkur et al., 2006). However, several studies have also shown that individual loci were not responsible on their own for specific anatomical and functional features of DS (Roper and Reeves, 2006; Shukkur et al., 2006).

Interestingly, the SUMO3 gene is located on the long arm of the Hsa21 and it was reported that there is an increase in SUMO3-modified proteins in the human hippocampus of post-mortem DS patient (Gardiner, 2006). This increase in SUMO3-sumoylation impacts a large number of target proteins that may include important molecular pathways involved in the synaptic function and disruption of their sumoylation/desumoylation balance may explain at least in part, some of the synaptic defect observed in the patients. Therefore it would be of great interest to identify and functionally characterize the increased SUMO3 target proteins in DS to evaluate whether this imbalanced sumoylation may account for some of the reported DS features.

### Parkinson’s Disease (PD)

PD is a neurodegenerative condition caused by impairments of striatal dopaminergic neurotransmission, and ultimately leads to gradual loss of dopaminergic neurons in the substantia nigra. Loss of these neuronal projections toward the striatum is directly correlated with the symptoms of the disease as the striatal structure is responsible for the control of voluntary movements. Accordingly, PD patients show significant decline in motor and non-motor functions, which are symptomatically expressed as resting tremor, muscle rigidness, impaired balance as well as speech and writing difficulties. Only 5% of the PD patients are diagnosed with genetic form of PD and the etiology of PD is yet to be fully elucidated. A cellular hallmark of the disease is the formation of intraneuronal inclusions known as Lewy bodies (LBs) that are often positive for SUMO1, ubiquitin and α-synuclein (for a recent review, see Vijayakumaran et al., 2015), therefore linking the SUMO pathway to the disease.

#### Sumoylation of α-Synuclein (αSYN)

The major component of LBs is αSYN, a small protein of 14 kDa encoded by the SNCA gene on chromosome 4. About 18 mutations in this gene have been directly linked to familial forms of PD and generally associate with the early-onset form of the disease, which typically appears before the age of 50.

The physiological functions of αSYN are still not clearly established but the protein is mainly localized at presynaptic sites where it is believed to regulate neurotransmitter release via its direct association to SNARE-proteins (reviewed in Calo et al., 2016). Structurally, αSYN contains an N-terminal membrane-binding domain, a hydrophobic core centrally involved in protein-protein aggregation, and an acidic C-terminal tail. Under physiological conditions, αSYN is able to fold into soluble tetra- and octameric protein structures. In LBs, αSYN misfolding leads to cytotoxic aggregates containing insoluble αSYN of high molecular weight species. It should be noted that recent experimental data showed that the intermediate oligomeric species of αSYN are toxic and most likely precede the formation of LBs in PD (Karpinar et al., 2009; Winner et al., 2011; Peelaerts et al., 2015).

αSYN was shown to be modified by SUMO1 and SUMO2 in cultured cells and in mammalian brain, and SUMO1 was also found in the brain of PD patients at the periphery of LBs co-localizing with αSYN, which raises the possibility that the SUMO pathway plays a role in protein aggregation (Dorval and Fraser, 2006; Kim et al., 2011; Krumova et al., 2011). Krumova et al. (2011) engineered a transgenic His-tagged SUMO2 mouse model and reported that sumoylation of αSYN occurs at the lysine 96 and 102 residues. They further showed that αSYN sumoylation reduces its propensity to aggregate in dopaminergic neurons of a rat model of PD. However, another study published almost simultaneously that sumoylation of αSYN promotes αSYN aggregates formation (Oh et al., 2011). Intriguingly, aggregates and inclusions formed as a result of impaired proteasome activity contain the sumoylated form of αSYN (Kim et al., 2011). Since the sumoylation of αSYN does not affect its ubiquitination, a proteasomal dysfunction may result in the accumulation of sumoylated αSYN and subsequently in αSYN toxic aggregation (Kim et al., 2011). Of note, a study that was performed in yeast confirmed the protective role of sumoylation against αSYN aggregation (Shahpasandzadeh et al., 2014). Moreover, this study showed that phosphorylation of αSYN can be additionally important for αSYN clearance through proteosomal degradation and suggested that sumoylation could modulate the interaction of αSYN with different kinases influencing its degradation (Shahpasandzadeh et al., 2014). Whether there is an active interplay between these two modifications of αSYN and whether they play a cell protective function against PD in the mammalian brain remains to be tested.

Interestingly, the extracellular spreading of αSYN has been reported in PD and Kunadt et al. (2015) have recently examined the possibility that sumoylation could serve as a regulatory mechanism for the sorting and the extracellular vesicular release of αSYN in neurons. They showed that sumoylation of proteins can mediate their extracellular sorting via the Endosomal Sorting Complex Required for Transport (ESCRT) into the extracellular vesicle pathway. Most importantly, they demonstrated that SUMO is recruited to ESCRT formation sites by the interaction with phosphoinositols and that sumoylation acts as a sorting signal for the extracellular vesicular release of αSYN (Kunadt et al., 2015). These data provide strong evidence for a role of the SUMO modification as a regulator of αSYN sorting to the extracellular space, possibly contributing to the interneuronal toxic spreading of αSYN reported in the disease and consequently to the etiology of PD.

#### Sumoylation of DJ-1

DJ-1 mutations have been linked to 1–2% of early-onset PD cases. DJ-1 is a molecular chaperone with cytoprotective functions under oxidative stress; in addition DJ-1 also acts as a transcriptional regulator. The DJ-1 protein is expressed in all brain regions, localizing to neurons and glial cells. DJ-1 is found within the cytoplasm, the nucleus, and in association with the mitochondria and the endoplasmic reticulum (reviewed in Eckermann, 2013). Interestingly, DJ-1 is present in presynaptic terminals, colocalizing with synaptophysin and associating with synaptic vesicles and also at the postsynapse, in dendritic spines (Usami et al., 2011) where it is involved in synaptic neurotransmission and induction of LTD (Wang et al., 2008).

Sumoylation of DJ-1 occurs on the lysine 130 residue and has been shown to increase upon UV irradiation. Moreover, this modification is necessary for DJ-1 to be in a fully activated form (Shinbo et al., 2006). The PD-associated DJ-1 mutation L166P leads to impaired DJ-1 sumoylation and decreases its solubility (Shinbo et al., 2006). Interestingly, the DJ-1 K130R mutation does not impact on the protein structure but rather leads to multi-/polysumoylation of the DJ-1 at alternative SUMO sites (Tao and Tong, 2003). Thus the elucidation of the exact synaptic function of DJ-1 sumoylation and how a defect in its sumoylation balance could impact synaptic function remains to be determined.

Overall, these findings highlight the key roles played by the sumoylation pathway in PD and we believe that the aim at clarifying the involvement of the SUMO process in the etiology of PD will become an active area of future research.

#### Sumoylation of Parkin

Although the loss-of-function mutations within the PARK2 gene, coding for the protein parkin, are the most common autosomal recessive juvenile causes of PD, the responsible molecular mechanisms remain unclear. Parkin is a RING-domain-containing E3 Ubiquitin ligase that is widely expressed throughout the CNS and can associate with PDZ scaffolding proteins at the postsynaptic membrane. Notably, parkin localizes to the majority of LBs in both familial and sporadic cases of PD. Recent findings suggest that parkin interacts with the KAR subunit GluK2 and regulates its neuronal function (Maraschi et al., 2014). Loss of parkin function, *in vitro* and *in vivo*, leads to GluK2 accumulation at the plasma membrane resulting in potentiated KAR current and consequently in the increase in KAR-dependent excitotoxicity presenting similar phenotype observed in autosomal recessive juvenile PD cases (Maraschi et al., 2014). Taking into account that GluK2 sumoylation regulates KAR endocytosis, neuronal excitability (Martin et al., 2007a) and synaptic plasticity (Chamberlain et al., 2012), it would be of high interest to see whether sumoylation could provide a rescue mechanism to down-regulate the increased surface expression and excitotoxicity seen in mouse brains that express the Parkin mutant causing autosomal recessive juvenile parkinsonism.

Importantly, parkin has also been shown to interact non-covalently with SUMO1 (Um and Chung, 2006). This interaction increased the E3 Ubiquitin ligase activity of parkin. Furthermore, it has been reported in this work that parkin specifically targets the SUMO E3 ligase RanBP2 for degradation (Um and Chung, 2006). Even though sumoylation of parkin or parkin substrates has not been directly involved in the pathogenesis of PD, it is reasonable to think, based on previous data, that sumoylation may directly impact on parkin’s function and so on the pathophysiology of PD.

### Huntington’s Disease (HD)

Unlike PD, HD has a monogenic fully penetrant cause with autosomal dominant inheritance. It belongs to the group of polyQ disorders that arise as a consequence of an expansion of the CAG trinucleotide repeat (encoding for glutamine) in specific genes. In HD, the deleterious CAG expansion leads to a polyQ expansion (≥ 40 instead of 23 glutamine residues in the normal Htt) within the amino-terminal domain of the Huntingtin (Htt) protein with the general agreement that longer polyQ expansions predict earlier onsets of the disease. Clinical hallmarks of HD are progressive motor decline leading to severe motor dysfunction, psychiatric disturbances and cognitive impairment. HD results from the toxic gain-of-function of expanded polyQ in Htt and its accumulation in affected neurons leads to neuronal cell death primarily in the striatum. Recent prevalence studies show that one individual in 7300 is affected in the western world (reviewed in Ross and Tabrizi, 2011).

#### Sumoylation of Huntingtin

Huntingtin is a large protein of 3144 amino acids (348 kDa) that folds into a superhelical structure with a hydrophobic core and serves as a scaffold protein. Htt is widely expressed in neurons and localizes both to the nucleus and the cytoplasm, shuttling between these two compartments. The cellular functions of Htt are still not well defined. Some studies suggested its roles in vesicular transport, regulation of gene transcription and RNA trafficking. Htt knockdown is lethal before the embryonic day 7.5 highlighting its critical role in embryonic development (Zeitlin et al., 1995). Htt indirectly interacts with NMDARs through PSD95 whereas presynaptic Htt is localized to synaptic vesicles, recycling endosomes and clathrin-coated vesicles (DiFiglia et al., 1995; Velier et al., 1998). Htt has been shown to influence the production and the transport of the growth factor BDNF in mice, and in cultured neurons Htt stimulates BDNF vesicle trafficking (Gauthier et al., 2004).

Importantly, several types of PTMs have been described for Htt including sumoylation and ubiquitination. A pathogenic fragment of Htt can be modified by both SUMO-1 and ubiquitin at the same lysine residue (Steffan et al., 2004). This group further showed that sumoylation stabilizes the pathogenic Htt fragment and reduces its ability to form aggregates in neuronal cell lines probably leading to a decrease in intracellular concentration of the toxic peptide (Steffan et al., 2004). Interestingly, genetic reduction of SUMO proteins in a *Drosophila* model of HD results in neuroprotection (Steffan et al., 2004). Moreover, potentiated sumoylation of mutant Htt that was caused by the action of the mutant Htt-specific SUMO E3 ligase Rhes, a striatal GTP-binding protein, displayed increased cytotoxicity (Subramaniam et al., 2009). Since sumoylation and ubiquitination of Htt occur on the same lysine residue and act in an antagonistic manner, it implies that the availability of the target lysine is critical for the degradation of Htt by the proteasome (Steffan et al., 2004). Because mutations that prevent these post-translational modifications on Htt reduce the pathology in *Drosophila*, it is likely that the balance between sumoylation and ubiquitination controls both the stability and the accurate targeting of Htt in neurons and that this tightly regulated balance is disrupted in HD.

More recently, O’Rourke et al. (2013) reported that Htt is sumoylated by both SUMO1 and SUMO2 primarily on the proximal lysine 6 and 9 residues and that PIAS1 is a SUMO E3 ligase for Htt. They further showed that genetic reduction of dPIAS in a mutant Htt *Drosophila* model of HD, which expresses mutant Htt, is protective confirming the previously reported positive role of sumoylation in HD (Steffan et al., 2004). The effect of Htt sumoylation by SUMO2 is the increase in the insoluble form of Htt in HeLa cells similar to the accumulation measured under proteasome inhibition. Importantly, this group also reported that the accumulation could be modulated by overexpression or acute knockdown of PIAS1 (O’Rourke et al., 2013). This supports the central role of the SUMO process in HD and also that a deregulated balance between sumoylation and desumoylation of Htt could participate in the etiology or in the aggravation of the disease. Accordingly, the authors reported an accumulation of SUMO2-modified proteins in insoluble fractions of HD post-mortem striata (O’Rourke et al., 2013).

### Alzheimer’s Disease (AD)

AD is the most common neurodegenerative condition causing severe memory deficits. AD accounts for more than 80% of dementia cases worldwide with no cure yet available (reviewed in Lee et al., 2013). Although the exact causes of AD are still much discussed, the pathology is characterized by the presence of intra- and extracellular protein aggregates mainly composed of Tau and β-amyloid (Aβ) proteins, which are toxic to the brain since they induce the loss of synapses, synaptic impairments and consequently, neuronal cell death (reviewed in Spires-Jones and Hyman, 2014).

Genetic studies have linked Single nucleotide polymorphisms (SNPs) in genes encoding the SUMO-activating enzyme SAE2 (Grupe et al., 2007; Corneveaux et al., 2010), the SUMO-conjugating enzyme Ubc9 (Ahn et al., 2009) and the desumoylase SENP3 (Weeraratna et al., 2007) to sporadic late onset AD. Immunohistological studies also revealed stronger SUMO immunoreactivities in hippocampal neurons of post-mortem AD brains compared to control patients (Li et al., 2003).

#### Sumoylation of Amyloid Precursor Protein (APP)

Interestingly, the APP from which Aβ is generated, and Tau have been both proposed to be substrates of the sumoylation machinery (reviewed in Spires-Jones and Hyman, 2014). Aβ is a small peptide of 4 kDa implicated in synaptic physiology and plasticity. The enzymatic machinery generating Aβ, which is composed of β- and γ-secretases, is partly localized in synaptic compartments. Aβ can directly bind to several synaptic receptors including NMDA and EphB2 receptors (De Felice et al., 2007; Simón et al., 2009). In cultured neurons the clustering of Aβ at excitatory synapses blocks the diffusion of mGlu5 receptors leading to increased calcium levels and hyperexcitability (Renner et al., 2010).

To date, the investigation into the effects of the sumoylation process in AD has generated mixed results. In 2003, the over-expression of SUMO3 was reported to dramatically reduce the Aβ production, whereas the expression of a SUMO3 form bearing the K11R mutation and therefore unable to form poly-SUMO chains displayed the opposite effect (Li et al., 2003). An additional work in 2008 showed that sumoylation of APP at the lysine 587 and 595 residues decreases the levels of Aβ aggregates in HeLa cells probably by altering the availability of the β-secretase cleavage (Zhang and Sarge, 2008). Conversely, two separate studies showed that sumoylation increases Aβ production independently of SUMO conjugation (Dorval et al., 2007) or via a mechanism involving the interaction of SUMO1 with BACE1, which is known to initiate the generation of Aβ (Yun et al., 2013). However, it should be noted that none of these studies examined the effects of Aβ sumoylation in neuronal cells. The authors rather used over-expression systems that might not reflect the exact mechanisms involved in the pathophysiology of AD. Considering the molecular complexity of AD and the off-target effects of SUMO over-expression, given the wide range of cellular pathway targeted by this modification, it will be of great interest to further address these discrepancies.

A more recent study examined the expression profile of the members of the SUMO machinery in the Tg2576 mouse model of AD that over-expresses APP (Nistico et al., 2014). They reported a significant increase in SUMO1-modified proteins and Ubc9 in the transgenic mice at 3 and 6 months of age compared to the WT littermates. SENP1 protein levels were also increased at the age of 3 months. On the contrary, the expression levels of SUMO2/3-modified proteins were markedly decreased at the age of 17 months and unchanged at the other examined stages (Nistico et al., 2014). This study thus supported the general belief of the field that the sumoylation/desumoylation balance is crucial and when deregulated it may participate in disease pathophysiology.

#### Sumoylation of Tau Protein

Tau is a microtubule-binding and stabilizing protein initially discovered to localize in axons (reviewed in Spires-Jones and Hyman, 2014). Recent data also suggest its roles in the regulation of protein composition at the postsynaptic density (Ittner et al., 2010). Moreover, tau was observed both in dendritic spines of normal as well as AD post-mortem brains (Tai et al., 2012). In AD, Tau is hyper-phosphorylated, detached from microtubules and aggregates into tangles within the somatodendritic region. It should be noted that not all neurons that have died display neurofibrillary tangles, suggesting that other pathological events must occur during the AD progression (Spires-Jones et al., 2014). Tau can be sumoylated in different cell types, mainly at the lysine 340 residue within a microtubule binding site (Dorval and Fraser, 2006). Tau sumoylation was affected upon proteasome inhibition suggesting that the lysine 340 residue is also a target for ubiquitination and that the balance between these two PTMs regulates Tau degradation (Dorval and Fraser, 2006). Intriguingly, the hyper-phosphorylated Tau aggregates stain positively for SUMO1 in an APP transgenic mouse model of AD, but not in post-mortem brain sections of AD patients (Pountney et al., 2003; Takahashi et al., 2008). In contrast, a recent study revealed that SUMO1 immunoreactivity colocalized with hyper-phosphorylated Tau in the cortex and hippocampal CA1 region of post-mortem AD brains, whereas no signal was measured for aged-matched control brains (Luo et al., 2014).

Sumoylation was also reported to promote Tau phosphorylation, and conversely, the hyper-phosphorylation of Tau induced Tau sumoylation (Luo et al., 2014). Luo et al. (2014) further showed that Tau sumoylation at the lysine 340 residue inhibited its ubiquitination and consequently its degradation. In addition, the exposure of cultured neurons to Aβ increased Tau-phosphorylation and sumoylation in a dose-dependent manner, which indicates that Aβ can act as an upstream regulator for tau phosphorylation and sumoylation (Luo et al., 2014). Altogether, these findings contribute to a better understanding of the role of sumoylation in AD and provide evidence for a putative mechanism explaining how the pathological accumulation of hyper-phosphorylated Tau occurs in AD brains.

## Concluding Remarks

It is nowadays very clear that the sumoylation process acts as a major signaling pathway essential for the regulation of synaptic function. The available set of identified sumoylated substrates is rapidly expanding at the presynaptic site but is still quite limited in the postsynaptic area. We are, however, confident that the recent technical advances in the proteomic field will allow the identification of novel SUMO target proteins and that such neuronal and synaptic SUMOylomes will help to better assess the central role fulfilled by sumoylation in synaptic transmission and plasticity.

As highlighted in this review, the sumoylation process contributes to a wide range of regulatory actions in the developing brain, and also that disruption of the equilibrium between the sumoylated and non-sumoylated state of proteins is directly linked to several neurodevelopmental and neurodegenerative diseases. Therefore, the better comprehension of the mechanisms that regulate the spatiotemporal distribution, targeting and activity of the sumoylation machinery in neurons will certainly provide valuable information regarding how the sumoylation/desumoylation balance is orchestrated in the brain and at synapses.

We can now expect that the functional characterization of novel SUMO regulatory pathways as well as the discovery of additional sumoylated substrates at all stages of the brain development will facilitate a deeper understanding of the SUMO process in brain function and help evaluate its potential implication in pathological conditions.

## Author Contributions

LS and SM wrote the manuscript. All authors listed, have made substantial, direct and intellectual contribution to the work, and approved it for publication.

## Conflict of Interest Statement

The authors declare that the research was conducted in the absence of any commercial or financial relationships that could be construed as a potential conflict of interest.
